# Complete Anticipation of Dr. Marshall Hall's Doctrine of "the Reflex Function," by Prochaska

**Published:** 1838-04

**Authors:** 


### COMPLETE ANTICIPATION OF DR. MARSHALL HALL'S DOCTRINE OF "THE REFLEX FUNCTION," BY PROCHASKA.

WE unaffectedly declare that we take shame to ourselves for having overlooked, in the composition of the article *on the Physiology of the Spinal Marrow*, in a preceding part of the present Number, the writings of PROCHASKA. Never, perhaps, was there a more remarkable anticipation of any doctrine supposed to be new than the anticipation of the doctrine of the so-called *Reflex Function* by the learned professor of Prague, as proved by the subjoined extract. This extract is taken from the third fasciculus of his *Annotationes Academicæ*, published at Prague in 1784. The anticipation of Prochaska differs from most of the other cases quoted in our article in this particular, that he does not notice the subject merely incidentally or as a matter of no importance: he clearly and explicitly announces the phenomena as depending on a distinct and important property of the nervous system, to which he devotes a whole chapter of his treatise. It is for this reason, and to avoid all possibility of cavil respecting the author's meaning, that we give the extract in the original,—and, for the present, without any translation. On reading this extract, we think even Dr. Hall will admit that nearly all that is defective in the views of Prochaska, respecting the doctrines of the reflex function, is a necessary consequence of his ignorance of the existence of the distinct properties of the roots of the spinal nerves, afterwards discovered by Sir Charles Bell. If we, as reviewers, confess ourselves ashamed of our previous ignorance of this portion of Prochaska's writings, we hope we may be forgiven for remarking that Dr. Hall's ignorance of it is still less pardonable.*

" CAPUT IV.—*Functiones Sensorii communis.*

§. I.—*Quid Sensorium commune, quæ ejus munia, & quæ sedes ?*

Impressiones externæ, quæ in nervos sensorios fiunt, per totam eorum longitudinem celerrime ad originem usque propagantur; quo ubi pervenerunt, reflectuntur certa lege, & in certos ac respondentes nervos motorios transeunt, per quos iterum celerrime usque ad musculos propagatæ motus certos ac determinatos excitant. Hic locus, in quo tanquam centro nervi tam sensui quam motui dicati concurrunt, ac communicant, & in quo impressiones nervorum sensoriorum reflectuntur in nervos motorios, vocatur, termino plerisque Physiologis jam recepto, *Sensorium commune.*

Non unus locus est, in quo sedem sensorii communis constituerunt Clariss. Viri. Bontekoe, Lancisius, de la Peyronie in corpus callosum posuerunt Sensorium commune; Willisius perceptionem sensuum & motus scaturiginem a corporibus striatis repetiit; Cartesius Glandulæ pineali munus sensorii communis tribuit; Vieussensius centro ovali; Boerhaave illud aggregatum punctorum pro Sensorio communi statuit, in quod omnes nervi sensorii terminantur, & ex quo omnes nervi motorii oriuntur, illudque ponit in medulla fornicata circumstante cavitatem ventriculorum†); in posteriore opere de morbis nervorum Boerhaavius Sensorium commune in confinio medullaris substantiæ cum corticali ponit, quam sententiam etiam verosimillimam esse autumatur Ill. Tissotus, & comprobari observatis Wepferi existimat‡); Cl. Mayer Sensorium commune in medulla oblongata ponere videtur§); hujus sententiæ quoque esse videtur Cl. Vir. I. D. Metzger||), Celeb. Camperus dicit, si Sensorium commune locum aliquem habet, illum debere esse in Glandula pineali, & natibus, testibusque, opinionemque Cartesii non esse adeo

* It is but justice to Professor Sharpey to state that we are indebted for our knowledge of the subjoined extract to him. Dr. S. noticed it recently in one of his lectures; and this was stated to us by a pupil.—ED.

† Prælect. acad. in proprias Inst. cum notis Halleri. Tom. iv. § 574.

‡ Abhandl. über Nerven und Nervenkrankh. 1ten Bands, 2ter Theil, § 236.

§ Abhandlung vom Gehirn, Rückenmark, &c. Seite 34, 38.

|| Advers. med., p. 15. Vermisch. Schrift. 1ten Bands, Seite 56.

ineptam*). Totum cerebrum cerebellumque certe non videtur ad Sensorium commune constituendum spectare, quæ partes systematis nervosi videntur potius instrumenta esse, quibus anima immediate utitur, ad actiones suas, animales dictas, peragendas; sed sensorium commune proprie dictum se per medullam oblongatam, crura cerebri cerebellique, etiam thalamorum opticorum partem, & totam medullam spinalem, verbo, quam late patet nervorum origo, extendere non improbabile utique videtur. Ad medullam spinalem usque Sensorium commune extendit†) docent motus in animalibus decapitatis superstites, qui sine nervorum ex medulla spinali oriundorum consensu ac commercio fieri non possent; nam rana decapitata si pungitur, non tantum punctam partem retrahit, verum etiam repit, & saltat, quod absque consensu nervorum sensoriorum & motoriorum fieri nequit, cujus consensus sedes in medulla spinali, superstite Sensorii communis parte, sit oportet.

Impressionum sensoriarum in motorias reflexio, quæ in Sensorio communi sit, non peragitur juxta solas leges physicas, ubi angulus reflexionis æqualis est angulo incidentiæ, & ubi, quanta fit actio, tanta etiam sequitur reactio; sed leges peculiares, a natura in pulpam medullarem sensorii quasi scriptas, sequitur ista reflexio, quas ex solis effectibus tantum noscere, neutiquam vero assequi ingenio nostro valemus. Generalis tamen lex, qua commune Sensorium impressiones sensorias in motorias reflectit, est nostri conservatio: ita ut impressiones externas corpori nostro nocituras sequantur certæ impressiones motoriæ, motus producturæ eo collimantes, ut nocumentum a corpore nostro arceatur, amoveaturque; & vice versa impressiones externas seu sensorias, nobis profuturas, sequantur impressiones internæ seu motoriæ, motus producturæ eo tendentes, ut gratus ille status ultro conservetur. Hanc generalem reflexionum Sensorii communis legem probant certe plurima exempla, quæ adduci possent, quorum pauca tantum adduxisse sufficiet. Irritatio in membrana narium interna facta excitat sternutationem, quia impressio illa ab irritatione in nervis olfactoriis facta per eos ad Sensorium commune defertur, ibi certa lege reflectitur in nervos motorios, musculis respirationi dicatis prospicientes, & per hos validam expirationem per nares producit, qua ab aëre vi transeunte irritamentum avellitur, & ejicitur. Ita fit, ubi irritatio in aspera arteria per micam cibi, vel guttulam potus illapsam causatur: facit hæc irritatio ad Sensorium commune delata, & ibidem in nervos respirationis motui dicatos reflexa, ut excitetur valida tussis, aptissimum ad expellendum irritamentum remedium, quæ prius non desinit, donec irritamentum ejectum non fuerit. Si amicus digito suo appropinquat ad oculum nostrum, licet persuasi simus nihil mali nobis inferendum esse, tamen jam impressio illa per opticum nervum ad Sensorium commune delata, in Sensorio ita reflectitur in nervos palpebrarum motui dicatos, ut nollentibus claudantur palpebræ, & arceant molestum digiti ad oculum attactum. Hæc & innumera, quæ afferri possent, exempla manifeste ostendunt, quantopere reflexio sensoriarum impressionum in motorias per Sensorium commune facienda conservationem nostri corporis respiciat. Propterea recte etiam Illustr. Tissotus actionem Sensorii communis illis viribus adnumerat, quarum summa atque connubium naturam corporis nostri viventis constituit‡).

Cum itaque præcipua functio Sensorii communis consistat in reflexione impressionum sensoriarum in motorias, notandum est, quod ista reflexio vel anima inscia, vel vero anima conscia fiat. Motus cordis, ventriculi, & intestinorum certe ab animæ conscientia nequaquam pendent, cum tamen nullus motus muscularis cieri possit, nisi stimulus nervis sensoriis applicatus in nervos motorios reflexione quadam transeat, & musculi contractionem cieat, tunc certum est reflexionem im-

* Kleine Schriften, &c. Leipzig, 1782. 1ter Band. Nachricht von der Zergliederung eines jungen Elephanten. § 21.

† Medullam spinalem ad Sensorium commune quoque referendam esse contendit Marherrus in Prælect. ad Instit. med. Boerhaavii, tom. ii. p. 404.

‡ Von Nerven, 2ten Bands, 2ter Theil, § 55, in nota prima. Et ibidem, § 6. Nro. 6. Qua de re etiam legere oportet Cl. Thaer, Diss. jam citatam de Actione de Systematis Nervosi in Febribus, & præprimis, § viii. ix. &c.

pressionum istis motibus excitandis aptarum, si in Sensorio communi fiunt, fieri sine animæ conscientia. Verum quæritur, utrum istæ impressiones ad Sensorium commune usque adscendant reflectendæ, an vero sine hac ambage citius in gangliis, unde plurimos nervos istæ partes habent, reflectantur? Hac de re postea adhuc agetur. Sed fieri tamen reflexiones impressionum sensoriarum in motorias in ipso Sensorio communi anima prorsus nescia docent actiones quædam in apoplecticis, quibus tota conscientia ablata est, superstites: nam & pulsu forti gaudent, & valide respirant, & etiam manum elevant, locoque affecto persæpe admovent inscii. Agit etiam Sensorium commune sine animæ conscientia convulsivos motus epilepticorum producendo, & etiam illas, quæ in profundo somno sepultis præter motum cordis & respirium aliquando observantur, artuum punctorum & leviter vellicatorum retractiones. Huc quoque spectant omnes motus, qui in corpore decapitati hominis aut alius animalis aliquo tempore supersunt, & vellicato corpore, præprimis vero medulla spinali, excitantur, qui certe sine conscientia animæ fiunt, & per residuam Sensorii communis partem, quæ in medulla spinali est, reguntur. Omnes istæ actiones ex organismo & physicis legibus, Sensorio communi propriis, fluunt, suntque propterea spontaneæ ac automaticæ. Illæ actiones quæ in corpore animali fiunt anima conscia, vel sunt tales, in quas anima voluntatis suæ nullum habet imperium vel vero tales, quas anima coercere & impedire pro libitu potest: illæ cum solo Sensorio communi, quatenus ab anima non dependet, regantur, etiam nihilominus, quam quæ inscia mente fiunt, automaticæ actiones sunt: tales est sternutatio a stimulo naribus applicito, tussis a stimulo traheæ illapso, vomitus a titillatione faucium aut emetico assumto, tremores ac convulsiones in chorea S. Viti, & in paroxismo febris intermittentis, &c. Actiones vero, quas anima suo imperio dirigit ac moderatur, quamvis etiam in iisdem producendis Sensorium commune suam partem habeat, vocamus nihilominus animales, non automaticas, de quibus capite sequente agetur."

---

